# Exosomal miRNAs in hepatocellular carcinoma development and clinical responses

**DOI:** 10.1186/s13045-018-0579-3

**Published:** 2018-04-11

**Authors:** Shuangshuang Li, Jiping Yao, Mingjie Xie, Yanning Liu, Min Zheng

**Affiliations:** Zhejiang University First Affiliated Hospital State Key Laboratory for Diagnosis and Treatment of Infectious Diseases,Clinical research center for hepatobiliary and pancreatic diseases of Zhejiang Province, Zhejiang University, Hangzhou, China

**Keywords:** Exosome, miRNAs, Hepatocellular carcinoma, Diagnosis

## Abstract

Hepatocellular carcinoma remains the sixth most lethal malignancy in the world. While HCC is often diagnosed via current biomarkers at a late stage, early detection of HCC has proven to be very difficult. Recent studies have focused on using exosomal miRNAs in clinical diagnostics and therapeutics, because they have improved stability in exosomes than as free miRNAs themselves. Exosomal miRNAs act through novel mechanisms for inducing cellular responses in a variety of biological circumstances. Dysregulated expression of miRNAs in exosomes can also accelerate HCC progression, including cell proliferation and metastasis, via alteration of a network of genes. Growing evidence demonstrates that exosomal miRNAs can affect many aspects of physiological and pathological conditions in HCC and indicates that miRNAs in exosomes can not only serve as sensitive biomarkers for cancer diagnostics and recurrence but can also potentially be used as therapeutics to target HCC progression. In this review, we summarize the latest findings between exosomal miRNAs and HCC, in order to better comprehend the functions and applications in HCC. Moreover, we discuss critical issues to consider when developing anti-tumor exosomal miRNAs as a novel therapeutic strategy for treating HCC in the clinic.

## Background

Hepatocellular carcinoma (HCC) is the sixth most lethal malignancy and ranks as having the third highest mortality in cancer-related death worldwide [1]. It shows a poor prognosis and a high risk of recurrence. Unfortunately, appropriate biomarkers for early HCC diagnosis are still lacking, and resection, liver transplant, or other chemotherapies for unresectable liver cancer remain the main choices for HCC therapy, even though the benefit is limited [2]. Therefore, it is urgent to identify effective, noninvasive, and specific biomarkers that provide early predictive potential in identifying HCC. Additionally, more biotherapies for HCC treatment are desperately needed. The growing interest in the role of exosomal miRNAs raises new questions about their role in HCC development and their potential as a therapeutic modality.

miRNAs are small noncoding RNAs containing about 19–22 nucleotides. miRNAs bind to the 3′UTR of pre-mRNAs during the process of post-transcriptional regulation and guide degradation of target mRNAs. Recently, with the rapid development of next-generation sequencing, miRNAs have been described more frequently in the diagnosis of malignant tumors. Therefore, miRNAs may play major roles in tumorigenesis, which would not even have been considered decades ago [3]. There have been many studies about the role and application of miRNAs in HCC [4, 5]. Interestingly, miRNAs are commonly found within exosomes. Exosomes are nanosize membrane-bound vesicles released from all types of cells, which are involved in intercellular communication [6]. This communication can be between donor cells and recipient cells. Exosomes provide a novel way to transfer effector messages between cells including RNA, proteins, miRNAs, long noncoding RNAs (lncRNAs), or DNA fragments [7, 8]. Although exosomes have been studied for many years, biological roles for exosomal miRNAs are just beginning to be understood, especially in HCC. Recently, some studies of exosomal miRNAs suggested that they might serve as more sensitive biomarkers for detection and treatment in HCC than simple miRNAs. Because of the much greater stability in exosomes than miRNAs themselves, they possess important biological properties that ensure long-term persistence.

## miRNAs and exosomal miRNAs

There are many similarities and differences between miRNAs and exosomal miRNAs in regard to their production, preparation, and purification. Both go through the same production process with miRNAs being transcribed into primary miRNAs via RNA polymerase II. Following Drosha processing, precursor miRNAs are formed, which are then transported into the cytoplasm through the exportin5 (XPO5) complex, and cleaved by Dicer to become mature miRNAs [9]. miRNAs that are packaged into exosomes are selective, and according to current studies, there are different views of this sorting mechanism. Since proteins of the RNA-induced silencing complex (RISC) have been detected in exosomes, it is plausible that the RISC modulates packaging efficiency of miRNAs in exosomes [10]. Other possible mechanisms include 3′ modification, association with target mRNAs [11], dependence on neural sphingomyelinase 2 (nSMase2) [12], or sumoylation by heterogeneous nuclear ribonucleoprotein (hnRNP)-related processes [13]. However, the precise mechanism of this sorting remains largely unknown.

Preparation and purification of miRNAs usually depends on the type of RNA extraction kit used. Samples can be obtained from body fluid including saliva, serum, and plasma. While extracting exosomal miRNAs is more complicated, the first step is to isolate exosomes by differential centrifugation and density-gradient centrifugation. The second step is to obtain miRNAs in exosomes through using an RNA extraction kit.

In recent years, exosomal miRNAs have been shown as important active component exosomes and this has attracted more and more attention. Exosomal miRNAs in body fluids and extracellular spaces have great potential to be taken up by other tumor cells or normal cells where they can exert functions. Dysregulation of exosomal miRNA expression has the potential to accelerate disease progression, through modulation of genome-wide gene networks. Recent research has revealed that exosomal miRNAs can affect many aspects of physiological and pathological conditions in liver cancer. Moreover, exosomal miRNAs can not only reflect the abnormal regulation in an intra-hepatic condition but also can influence other organs and their microenvironment. Accumulating evidence indicates that exosomal miRNAs are important molecules with significant function in HCC and that more research is needed in this area. In this review, we mainly summarize the major roles for exosomal miRNAs in HCC and put forward views on how to make use of these for future therapeutic application.

## Exosomal miRNAs in HCC development

Exosomal miRNAs play a critical role in mediating the HCC progress and metastasis signaling networks. The following sections will discuss the specific roles for exosomal miRNAs in major aspects of HCC development, such as cell proliferation, metastasis, immune escape, and interaction with the tumor microenvironment. Table 1 summarizes exosomal miRNAs in HCC development.

**Table 1:** Exosomal miRNAs in HCC development

Exosomal miRNAs	Donor cells	Recipient cells	Target genes	Functions	Ref.
miR-122	Huh7 cells	HepG2 cells	CAT1, FTF2B, etc.	Inhibit growth and proliferation and increase senescence of HepG2 cells	[16]
miR-122	AMSCs	HepG2 cells	ADAM10, IGF1R, CCNG1	Render HepG2 cells more sensitive to chemotherapeutic agents and inhibit HCC cells proliferation	[43]
miR-9-3p	^a^	^a^	HBGF-5	Reduce HCC cell viability and proliferation and reduce expression of ERK1/2	[36]
miR-335-5p	LX2 cells	MHCC97L, MHCC97H, Huh7, and HepG2 cells	CDC42, TCF3, CDK2, etc.	Inhibit recipient cell proliferation and invasion and reduce HCC tumor in size	[18]
miR-1247-3p	LM3 cells	Normal fibroblasts in lung tissues	B4GALT3	Promote the conversion of normal fibroblasts to CAFs and accelerate lung metastasis of HCC	[25]
miR-320a	CAFs	MHCC97-H cells and SMMC-7721 cells	PBX3	Inhibit tumor progression by suppressing MAPK pathway	[27]
miR-142, miR-223	Macrophages	Huh7 cells	STMN1	Inhibit HCC cell proliferation	[28]
miR-490	Mast cells	HepG2 and Hep3B cells	ERGIC3	Suppress EGFR/AKT/ERK1/2 pathway and inhibit migration of HCC cells	[20]
miR-584, miR-517c, miR-378, etc.	Hep3B cells	HepG2 cells	TAK1, TAB2, etc.	Associate with hepatocarcinogenesis and enhance transformed cell growth in recipient cells	[21]
miR-155	Arsenite-transformed L-02 cells	Native L-02 and THLE-3 cells	QKI	Promote inflammatory infiltration and HCC	[24]

^a^Exosomal miR-9-3p is from serum in this research, so no donor cells and recipient cells are mentioned

### Exosomal miRNAs in HCC growth and metastasis

miR-122 is a liver-specific miRNA and the most frequent miRNA in the adult liver where it can inhibit HCC cell growth [14, 15], and also can be transferred by exosomes between cells. When Huh7 cells and HepG2 cells are growing in co-culture, miR-122 is released from Huh7 cells within exosomes, which could be absorbed by miR-122-deficient HepG2 cells. This miR-122 transfer was sufficient to repress target mRNAs and inhibit growth to promote senescence of recipient HepG2 cells. Furthermore, a reciprocal interaction between the two cell lines was observed where HepG2 cells could excrete insulin-like growth factor 1 (IGF-1) to decrease miR-122 expression in Huh7 cells in order to insure its own proliferation. This level of cross-talk between cells is extremely interesting and suggests that there is much more that we need to learn about this level of intercellular communication. This may be a key mechanism to balance the growth between cells and the microenvironment [16]. Exosomal miR-9-3p has also been reported to suppress expression of fibroblast growth factor 5 (HBGF-5), to play a vital role in cell proliferation, and to restrain development of HCC [17].

Recently, a study uncovered a novel interaction between human hepatic stellate cells (LX2) and HCC cells (MHCC97L, MHCC97H, Huh7, and HepG2 cells) by exosomal miR-335-5p secreted from LX2 cells. Further investigations have shown that over-expression of miR-335-5p in exosomes from LX2 could be absorbed by recipient HCC calls to inhibit HCC cell proliferation in vitro and suppress the tumor growth in vivo. Interestingly, downregulation of miR-335-5p within these fibroblasts as well as in HCC cells was observed, when co-culturing LX2 with any of the four HCC cell lines, respectively. These findings demonstrate that stromal cells can provide a more suitable environment for HCC growth, providing a rich network of intercellular connections between cirrhotic/fibrotic liver and HCC, thus validating the significant role of exosomal miRNAs in transforming liver cirrhosis to HCC [18].

Metastasis is generally the leading cause of cancer-related death, especially in HCC. HCC is characterized by a tendency for multifocality and dysregulation of multiple signaling pathways adding to the complexity of the disease. Our previous data showed that adding exosomes from high-metastatic HCC cells (MHCC97H) to low-metastatic HCC cells (HepG2) has a great effect by enhancing migration ability of the HepG2 cells. Interestingly, miRNAs in the exosomes were very different within two types of cells, suggesting that unique miRNAs in exosomes have unique functions in HCC cells. This was confirmed in another study showing interactions between HCC cells and normal hepatocytes. The article revealed that exosomes derived from motile HCC cell lines carrying a large number of noncoding RNAs including miRNAs could significantly improve the migration and invasion capacity of nonmotile MIHA (an immortalized hepatocyte) line cell [19].

There are numerous other examples of this type of exchange of motility ability from one cell type to another. For example, exosomal miR-335-5p produced by LX2 cells could decrease migration and invasion of HCC cells [18]. Mast cells stimulated by hepatitis C virus E2 envelope glycoprotein could transfer exosomal miR-490 to HCC cells and restrain the ERK1/2 pathway to inhibit HCC cell metastasis. These results provide a brand new perspective toward biological therapy of HCC and hepatitis C [20]. In another study, exosomal miRNAs from HCC cells could regulate the expression of transforming growth factor-β-activated kinase-1 (TAK1) and promote growth and invasion of transformed cells in recipient HCC cells [21]. Altogether, these observations demonstrate a unique intercellular mechanism, mediated by exosomal miRNAs, capable of contributing to the local spread through promoting intra-hepatic metastases or multi-focal growth in HCC.

### Exosomal miRNAs and the tumor microenvironment

Tumor development is a multi-stage process where normal cells obtain characteristics that ultimately lead to their conversion into cancer cells [22]. A complex tumor microenvironment contributes to tumor progression by intercellular communication between malignant cells and normal cells [23]. Exosomal miRNAs play a vital role in HCC through recruitment of inflammatory infiltration and stimulation of the immune response. miR-155 is known to be involved in inflammation and has been verified as transferrable by exosomes that link with other types of cells to execute biological functions. miR-155 from arsenite-transformed human hepatic epithelial cells (L-02) could be assimilated by normal L-02 and THLE-3 cells (human liver epithelioid cells) via exosomes and induce a pro-inflammatory phenotype in normal recipient liver cells through higher expression of IL-6 and IL-8. These cytokines, especially IL-6, activate p-STAT3 and provide a strong association with arsenite-induced carcinogenesis. Environmental chemicals could induce exosomal miRNAs to modulate inflammatory infiltration from pathological cells to normal ones [24].

The formation of a pre-metastatic niche also plays a very important role in tumor progression. The microenvironment of the metastatic niche, which provides support for tumor cells, can lead to distant metastases. A recent study demonstrated that exosomal miR-1247-3p from HCC cells, especially from high-metastatic HCC cells, could promote lung metastatic niche formation, thus facilitating conversion and activation of cancer-associated fibroblasts (CAFs). This conversion occurs through activation of β1-integrin–NFκB signaling in normal fibroblasts, which accelerates lung metastasis of liver cancer cells. Furthermore, the level of serum exosomal miR-1247-3p is positively correlated with the outcome of lung metastasis in HCC patients [25].

Fibroblasts are associated with cancer in all processes of disease development. As a considerable component of the tumor microenvironment, CAFs produce and secrete many extracellular matrix molecules, including pro-inflammatory cytokines, that contribute to cancer progression [26]. Many studies have demonstrated that dysregulation of exosomal miRNAs promote the production and activation of CAFs. Activation of CAFs exerts a big role in HCC, and one of the interactions between CAFs and HCC cells has been investigated. Zhang and his colleagues found significant reduction of miR-320a level in CAF-derived exosomes compared with exosomes from matched para-cancer fibroblasts in HCC. This group deduced that downregulated expression of miR-320a in exosomes derived from CAFs contributed to the HCC malignant phenotype transition, showing the CAF-mediated HCC tumor progression is partially related to loss of anti-tumor miR-320a in the exosomes of CAFs [27].

Furthermore, the roles of exosomal miRNAs should not be ignored in immune environment. When co-cultured with macrophages, expression of miR-142 and miR-223 increased dramatically in Huh7 cells and inhibited HCC cell growth. Further investigation revealed that this phenomenon occurs through exosomes. Therefore, macrophages release exosomal miRNAs to suppress HCC development [28].

The microenvironment in specific organs also communicates with metastatic cells through exosomal miRNAs. A recent study revealed that brain astrocyte-derived exosomes mediated an intercellular transfer of PTEN-targeting miRNAs (principally miR-17-92 cluster) to metastatic tumor cells and led to downregulation of PTEN mRNA and protein. This miRNA transfer ultimately facilitated tumor metastasis, and the phenomenon was reversible once the metastatic tumor cells destroyed the brain microenvironment. This interesting finding demonstrated an essential role of reciprocal cross-talk by exosomal miRNAs between the metastatic cells and the organ-specific metastatic niche. More importantly, this study provided new insights toward effective anti-metastasis treatments, especially for HCC patients or perhaps those with other cancers with distant organ metastasis [29].

## Exosomal miRNAs for diagnosis, prognosis, and warning of recurrence in HCC

Most HCC patients are diagnosed at a very late stage because accuracy of common indicators such as AFP in the early diagnosis of HCC is not sufficient [30]. Hence, novel noninvasive biomarkers with high sensitivity and specificity for early-stage HCC diagnosis are imminently needed, especially for high-risk patients. Since miRNAs in exosomes prevent degradation from RNase activity due to protection of the lipid bilayer, miRNAs are more stable in serum exosomes [31]. Therefore, application of exosomal miRNAs as potential biomarkers for diagnosing HCC is warranted. As noninvasive biomarkers, the roles of serum exosomal miRNAs for diagnosis and for assessing prognosis of HCC have been under active investigation. Table 2 summarizes the clinical significance of exosomal miRNAs in HCC. Upregulated and downregulated expression of exosomal miRNAs are both possible under pathological circumstances. There are no fixed principles to use for distinguishing their role. Therefore, researchers should give extra vigilance to miRNA levels, regardless of whether they are high or low.

**Table 2:** Clinical significance of exosomal miRNAs in HCC

Clinical significance	Exosomal miRNAs	Source	Expression in HCC	Clinical relevance	Ref.
Detection and diagnosis	miR-665	Serum	Up	Higher in HCC than the healthy and associated with HCC progression	[32]
	miR-18a, miR-221, miR-222, miR-224	Serum	Up	Distinguishes HCC from CHB or LC	[34]
	miR-21	Serum	Up	Higher in HCC than in CHB and in healthy volunteers	[35]
	miR-101, miR-106b, miR-122, miR-195	Serum	Down	Distinguishes HCC from CHB or LC	[34]
	miR-9-3p	Serum	Down	Lower levels in HCC than in healthy donors	[36]
Detection, prognosis and recurrence	miR-638	Serum	Down	Predicts poor prognosis in HCC patients	[33]
	miR-125b	Serum	Down	Lower levels of exosomal miR-125b correlated with shorter time to recurrence and reduced overall survival	[38]
	miR-718	Serum	Down	Poor prognosis and high rate of recurrence after liver transplantation	[37]
Potential treatment	miR-122	Cells	Down	Renders HCC cells more sensitive to chemotherapeutic agents and improves the anti-tumor effect of sorafenib on HCC in vivo	[43]
	miR-335-5p	Cells	Down	Inhibits recipient cell proliferation and tumor growth in vivo	[18]

Exosomal miRNAs as biomarkers can be used for detection, diagnosis, prognosis, and recurrence and can be a potential treatment of HCC

*HCC* hepatocellular carcinoma, *CHB* chronic hepatitis, *LC* liver cirrhosis

Recently, a study revealed that HCC patients had obviously higher serum exosomal miR-665 levels than healthy subjects. In these patients, serum exosomal miR-665 level was closely related to tumor size, invasion, clinical stage, and survival time. Therefore, the higher the level of serum exosomal miR-665, the worse the clinical situation; this suggests that serum exosomal miR-665 could be a suitable noninvasive biomarker for diagnosis and prognosis in HCC [32]. Additionally, another study has shown that loss of miR-638 is negatively associated with tumor size, vascular invasion, and disease stage, and predicts poor prognosis in HCC patients [33].

Another study showed serum levels of exosomal miR-18a, miR-222, miR-221, and miR-224 were dramatically higher in patients with HCC than in those with chronic hepatitis B (CHB) or liver cirrhosis (LC). While the levels of serum exosomal miR-101, miR-106b, miR-122, and miR-195 were significantly lower in HCC than in CHB, among the three groups, there was no obvious difference in the levels of miR-21 and miR-93. Additionally, expression of serum circulating miRNAs, miR-221, miR-222, and miR-224, between HCC and LC groups was similar to that of serum exosomal miRNAs; however, the difference of serum miRNA levels was smaller than those in serum exosomes. Furthermore, unlike exosomal miRNAs, serum-circulating miRNAs did not show an obvious difference between CHB and HCC groups. These findings indicated that serum exosomal miRNAs were better for distinguishing HCC from CHB or LC compared with the level of serum circulating miRNAs [34].

While the Wang group demonstrated a different view of exosomal miR-21, his group found that serum exosomal miR-21 level was much higher in HCC than in CHB or in healthy controls. Furthermore, there was a close correlation between serum exosomal miR-21 level and cirrhosis or advanced tumor stage. Otherwise, although serum-circulating miR-21 had an analogous result, the sensitivity of detection was inferior to serum exosomal miR-21. The data demonstrate that serum exosomal miR-21 can be a novel indicator with more sensitivity than whole serum in HCC [35]. Another study pointed out that serum exosomes from patients with HCC contained remarkably lower levels of the miR-9-3p than did serum exosomes from normal donors, also suggesting a potential role for this miRNA in HCC [36].

The problem of poor prognosis and frequent recurrence in HCC is also troubling. HCC patients with recurrence after liver transplantation have lower serum exosomal miR-718 than those without recurrence. A mechanism of action involving loss of miR-718 and increased expression of HOXB8 was linked with the poor prognosis [37]. The result suggested potential benefit for selecting HCC patients with better LT adaptation. Another study showed serum exosomal miR-125b could be a highly accurate and effective biomarker for recurrence and survival time in HCC patients after liver resection. The research revealed exosomal miR-125b levels were higher than that in the whole serum or supernatant without exosomes among CHB, LC, and HCC patients. Furthermore, compared with CHB and LC groups, serum exosomal miR-125b levels were lower in HCC group, and the cases with lower levels of exosomal miR-125b had a shorter time of recurrence and overall survival [38].

As mentioned above, exosomal miRNAs are of great use in HCC diagnosis and prognosis and may predict recurrence. However, we also acknowledge that different studies have had different views even regarding the same exosomal miRNA. This may be in part due to the number of the patients included in the studies not being sufficient. Furthermore, individual differences between patients can be considerable and subsequent bioinformatics after detection may also vary. When analyzing the differential expression of exosomal miRNAs, researchers must consider various factors, such as age, gender, virus infection, and the accurate process of disease. In the future, more samples of all kinds of stages of HCC should be included to improve the reliability of determinations of serum exosomal miRNA and to increase their usefulness as robust and accurate biomarkers.

## Exosomal miRNAs for use in HCC therapeutic strategies

Recently, a large number of studies have suggested that exosomal miRNAs could present an attractive new vehicle for cancer therapy. For example, exosomes from mesenchymal stem cells (MSCs) carrying miR-124a were effective in treating glioblastoma [39], and exosomal miR-214-3p from osteoclast was able to suppress osteoblastic bone formation [40]. In contrast, tumor-promoting miRNAs in exosomes have a reserve effect. Ovarian cancer cells obtained paclitaxel resistance from stroma-derived exosomal miR-21 and then promoted tumor development [41]. Similarly, exosomal miRNAs in HCC promote tumorigenesis. Therefore, novel therapeutic strategies can be envisioned given the proper treatment regimen. Here, we discuss several approaches that have already been tested.

### Exosome-mediated miRNA transfer for HCC therapy

**Fig. 1:**
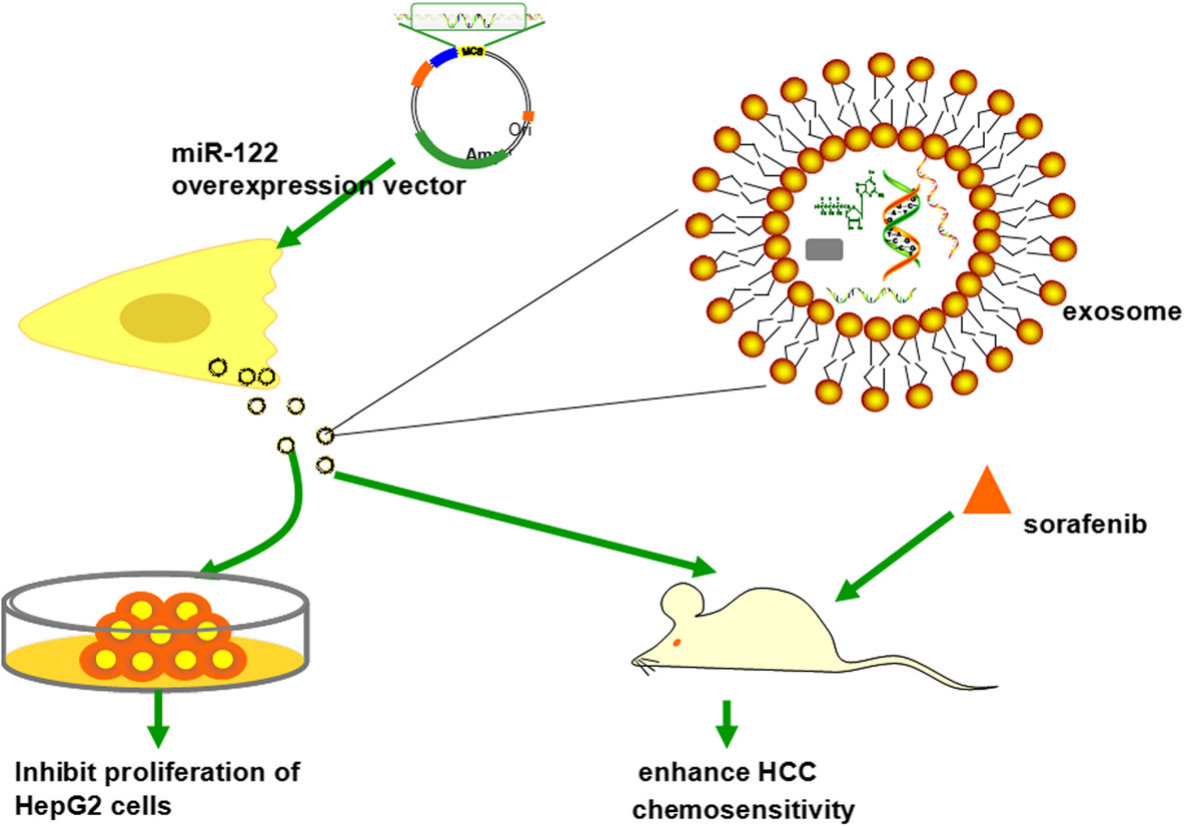
Exosomal miR-122 from adipose tissue-derived mesenchymal stem cells (AMSCs) could inhibit the proliferation of recipient HepG2 cells and enhance HCC chemosensitivity in vivo. This diagram shows the experimental platform that has been tested using HepG2 cells to modulate their sensitivity to chemotherapeutics

Exosomal miRNAs are a potentially useful tool against HCC, and they come with a theoretically reduced potential for adverse side effects. Exosome delivery as a shuttle for therapeutic miRNAs has previously been investigated and summarized in Fig. 1. Adipose tissue-derived mesenchymal stem cells (AMSC) used to produce exosomes result in very productive effects [42]. To begin, we transfected AMSC with a miR-122 over-expression plasmid, and then, the AMSC-derived exosomes (exo-122) were collected and added to HepG2 cells. Strikingly, we found that the AMSC-derived exosomes were enriched for miR-122 and rendered HepG2 cells more sensitive to chemotherapeutic agents through miR-122 transfer from AMSCs to recipient HepG2 cells. Furthermore, injection of exosomal miR-122 improved the anti-tumor effect of sorafenib on HCC in vivo [43].

miR-122 is a key factor involved in liver development and metabolic function. Loss of miR-122 is associated with various forms of liver diseases which can be restored by miR-122 expression. miR-122 can also modulate HCC in vivo or in vitro [14, 15, 44, 45]. Therefore, in total, our data demonstrate that exosomal miR-122 has a novel ability to enhance HCC chemosensitivity, thereby providing a new treatment strategy for HCC [43]. As previously mentioned, stellate cell-derived exosomal miRNAs such as miR-335-5p could be delivered in vivo and serve as a novel therapy in HCC [18]. Another example is that promoting the transfer of CAF-derived miR-320a might be a potential treatment option to overcome HCC progression [27].

In addition to transfer of exosomal tumor-suppressive miRNAs to HCC tumor cells directly, we can also choose the tumor environment to be the object. Specifically, CAFs, immune cells, or tumor endothelial cells can all be regulated by exosomal miRNAs. T cell-derived exosomal miRNAs (mainly miR-92a) can be functional in antigen-presenting cells to promote the immune response [46]. Let-7d-containing exosomes from Treg cells transferred to Th1 cells promoted longevity, which documents an important regulatory role by exosomal miRNAs [47]. So exosomal miRNAs may be potential immunotherapy in HCC. All these findings demonstrate that exosomal miRNAs are promising and may become effective remedies for HCC in the future.

### Questions regarding therapeutic application of exosomal miRNAs

**Fig. 2:**
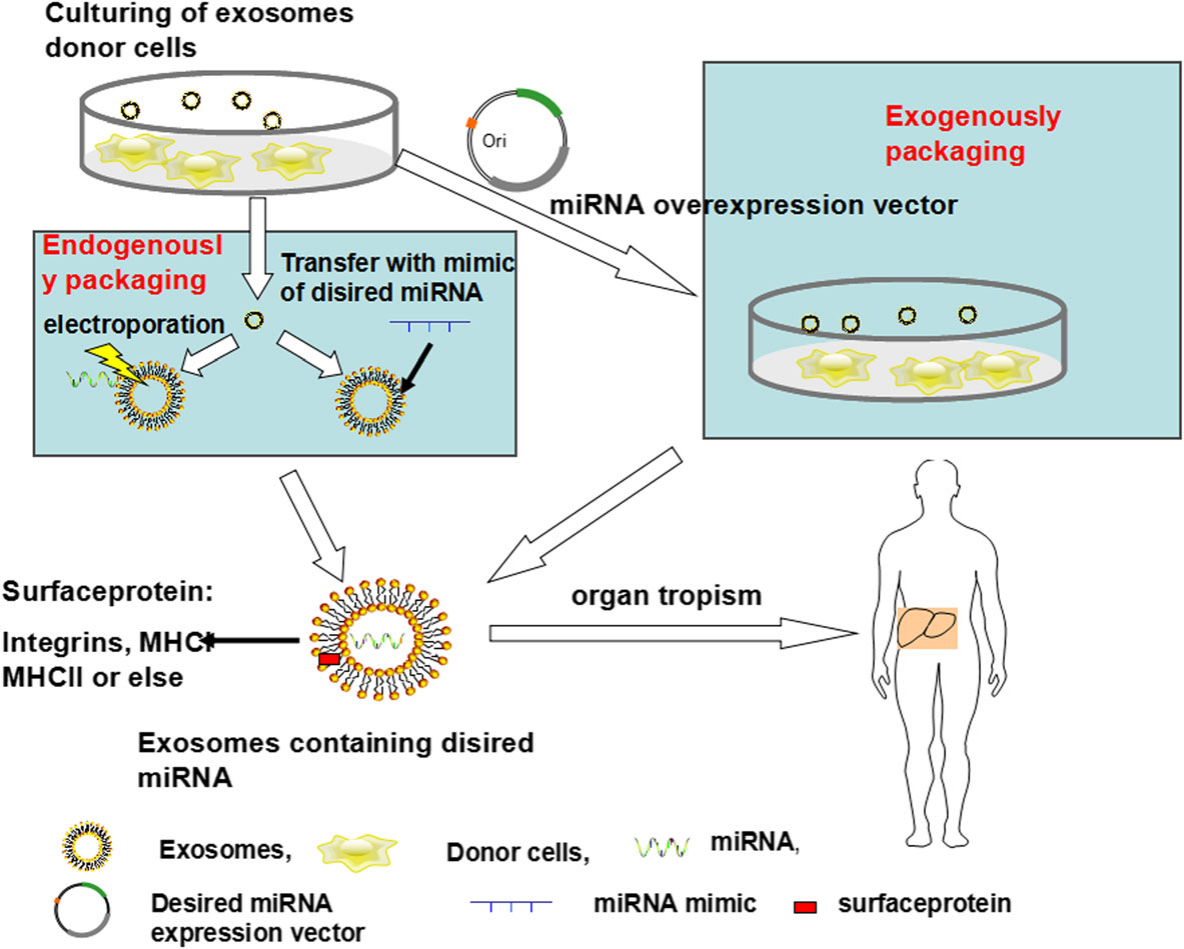
The process of miRNA-mediated treatment envisioned for future application in HCC patients. Based on experimental data generated using cell lines, a comparable approach for engineering exosomes containing miRNA cocktails can be tailored to the patient-specific needs. This approach has many technical hurdles to overcome but has great promise as future therapy for HCC with fewer side effects. Selecting and culturing appropriate cells to produce enough exosomes is a first priority. Second, endogenous and exogenous packaging of desired miRNAs into exosomes is important. The former would need to be done by transfecting miRNA over-expressing vectors into donor cells, while the latter would require transferring miRNA mimics into isolated exosomes or via electroporation. Transporting exosomes to the liver by intra-venous delivery or direct intra-tumoral injection is also a major objective

Although exosomal tumor-suppressive miRNAs are a promising avenue for new treatment strategies for HCC and other cancers, there are many issues that should be paid attention to (Fig. 2).

When it comes to the design of exosomal miRNA-mediated treatment strategies, exosomes should be harvested in sufficient amounts to provide therapeutic benefit. Therefore, exactly how to obtain the highest quality and most enriched exosomes from cells may require optimization. If the production parameters can be optimized, then the secretion mechanisms of exosomes can be leverage for therapy. We envision using some cells as a bio-factory to produce abundant exosomes. This type of advance in the production method involving isolation of exosomes from conditioned medium need to be developed. Ultracentrifugation is currently the most popular method, but it is time-consuming and lacks a uniformed standard. Moreover, reagents for isolation of exosomes are expensive. So establishing more cost-effective and time-saving isolation and purification methods is essential.

Incorporating the desired miRNA into exosomes remains a challenge. As described above, transfer of donor cells with miRNAs over-expressed from plasmids is one approach. We can also transfer a miRNA mimic to the isolated exosomes [18] using the same approach or alternatively through electroporation [48]. More effective methods are needed to improve the efficacy of this incorporation step.

The mechanism of miRNA secretion and uptake is not well defined. In particular, the mechanism by which tumor-suppressive miRNAs are sorted into exosomes is unknown. A major goal for therapy would be to make sure that only the desired miRNAs but not other miRNAs are packaged into exosomes since specificity for their activity is critical. Incorporation of miRNAs into exosomes occurs in a ceramide-dependent manner independent of the ESCRT (the endosomal sorting complexes required for transport) machinery [10]. Therefore, accumulating evidence has demonstrated that packaging of specific miRNA population into exosomes is selective [21, 49, 50]. Better understanding the mechanisms controlling this sorting behavior is very high priority in the field.

Deep sequencing of RNA from immune or tumor cell-derived exosomes has revealed the miRNA composition in exosomes and shown that it is not a mere reflection of the cellular miRNA but rather a specific series of miRNAs. In HCC, Vps4A which was associated with endosomal transport function in cell biology is regarded as tumor suppressor. It can increase secretion of oncogenic miRNAs (miR-27b-3p and miR-92a-3p) in exosomes as well as gather the tumor suppressor miRNAs (miR-193a-3p, miR-320a, and miR-132-3p) in HCC cells (SMMC-7721 cells) [51]. A similar study demonstrated that in human advanced colon disease, higher levels of tumor suppressor miRNA-193a accumulated in the exosomes. Further investigation revealed that knockout of the major vault protein led to miR-193a increase in the donor cells, not in exosomes, suppressing tumor progression [52]. Therefore, there is still a long way to go to understand the mechanism of selective packaging of miRNAs to exosomes.

Another key question yet to be solved is how to improve the ability of recipient cells to uptake the exosomes containing desired miRNAs. A relevant research study showed that in SMMC-7721 cells transfected with Vps4A-over-expressed plasmid, six tumor suppressor miRNAs were strikingly upregulated. After treatment with exosomes from SMMC-7721 cells, Vps4A increased the uptake of tumor suppressor miRNAs by exosomes in HCC cells. However, the mechanism by which Vps4A modulated exosome activity and the recipient cell response to exosomes requires further investigation [51]. Other possible molecules having similar functions may be responsible and should be investigated.

The immunogenicity of exosomes is still poorly understood. Many studies show that exosomes from self-derived cells have less immunogenicity than virus or other delivery methods [53]. Relevant studies about the reciprocity between HCC cell-derived exosomal miRNAs and immune cells have not been investigated. Further studies need to be done in the future.

Finally, the organotropism of exosomes also should be a point of concern. More and more studies have revealed that exosomes have a tropism for target cells and exosomes with integrins α6β4 and α6β1 tend to promote lung metastasis, while exosomes with integrin αvβ5 are associated with liver metastasis [54]. So in order to send the exosomes containing desired miRNAs to liver lesions accurately, we must understand the organotropism to ensure the correct destination of exosomes.

In summary, exploiting natural mechanisms of miRNA-mediated HCC tumorigenesis or other cancers is a promising approach but requires additional clarification of many steps. It is becoming clear that exosomes can absorb tumor-suppressed miRNAs and that this can be engineered to make sure that therapeutic exosomes can be transported and taken up by the designated tissues or organs. The liver plays a major role in this uptake and presents with the least potential side effects. Additional studies must identify the best delivery method to HCC patients for exosomes containing specific miRNAs. This may be via intra-venous delivery or, as in the case of TACE (Transhepatic Arterial Chemotherapy and Embolization), via direct delivery to the cancer mass. Moreover, there is no valid data to support the safety of these reagents, so careful testing in vivo is needed. Optimized methods for isolating exosomes and cultivation of donor cells will be necessary to facilitate clinical applications in humans.

To date, there have been only three phase I clinical trials conducted using therapeutic exosomes through an immune vaccine for cancer therapy, but none of these trials used exosomal miRNA-directed therapy. Therefore, there is still a long way to go before established exosomal miRNA-mediated treatments are available for HCC [55].

## Conclusions

In this review, we have summarized the current status of knowledge regarding the functions of exosomal miRNAs in HCC development and their clinical significance. We acknowledge that there are still many gaps in this research field that need to be filled. For example, the roles of exosomal miRNAs in regard to angiogenesis and hypoxia during EMT in HCC have not been investigated. Therefore, future work should shed more light on these research areas and illuminate a more comprehensive understanding of the mechanisms by which exosomal miRNAs have effector functions in HCC. On the other hand, we want to emphasize that tumor-suppressive exosomal miRNA-mediated cure is promising for HCC and other cancers, although several difficulties must be dealt with. The work required to harness the potential of this new therapeutic modality may prove to be a helpful remedy in HCC patients in the future.

## Abbreviations

AMSC: Adipose tissue-derived mesenchymal stem cells
CAFs: Cancer-associated fibroblasts
CHB: Chronic hepatitis
ESCRT: The endosomal sorting complexes required for transport
HBGF-5: Fibroblast growth factor 5
HCC: Hepatocellular carcinoma
hnRNP: Heterogeneous nuclear ribonucleoprotein
IGF-1: Insulin-like growth factor 1
LC: Liver cirrhosis
MSCs: Mesenchymal stem cells
nSMase2: Neural sphingomyelinase 2
RISC: RNA-induced silencing complex
TACE: Transhepatic Arterial Chemotherapy and Embolization
TAK1: Transforming growth factor-β-activated kinase-1
XPO5: Exportin5
